# Computational Simulation of Entropy Generation in a Combustion Chamber Using a Single Burner

**DOI:** 10.3390/e20120922

**Published:** 2018-12-03

**Authors:** Souad Morsli, Amina Sabeur, Mohammed El Ganaoui, Harry Ramenah

**Affiliations:** 1 Laboratoire des Sciences et Ingénierie Maritimes (LSIM), Faculté de Génie Mécanique, Université des Sciences et de la Technologie Mohamed BOUDIAF d’Oran, el M’Naouer, Oran BP 1505, Algeria; 2 Laboratoire de Conception, Optimisation et Modélisation des Systèmes (LCOMS), University of Lorraine, 57070 Metz, France; 3 Energetic Laboratory of Longwy, (FJV/LERMAB), Henri Poincaré Institute of Longwy, University of Lorraine, 54400 Cosnes et Romain, France

**Keywords:** diffusion flames, burner, entropy generation, Fluent, carbon monoxide emission

## Abstract

In this study, we examine the behavior of a propane diffusion flame with air in a burner; the computational investigations are achieved for each case employing the Fluent package. The graphs generated illustrate the influence of flow parameters, the effects of the oxygen percentage in the air, and the effects of the equivalence ratio *φ* on the entropy generation, the temperature gradients, and the Bejan number. The obtained results show that incorporation of hydrogen with propane reduced both temperature and carbon monoxide emission.

## 1. Introduction

Over the past two centuries, combustion processes have played an important role in industrialization, and as a result, the use of combustion processes in industry has increased exponentially due to the development of fossil fuel combustion. Currently, about 87% of the world’s primary energy still relies on fossil fuels [1]. Research on the optimization of combustion energy production systems focuses on improving energy efficiency in order to better manage the energy produced, and on reducing the environmental impact of combustion, characterizing the production of pollutants, such as nitrogen oxides, soot, and carbon monoxide. The reduction of carbon dioxide emissions is associated with energy optimization and the use of alternative fuels. Each type of energy is formed by the product of an intensive and an extensive variable, these being temperature and entropy. The general expression of the entropy variation of any system is related to entropic exchanges with thermal sources, and internal and external irreversibilities. The energy converters are the engines and the thermodynamic generators. The second principle then appears as the possibility of operating a heat generator with a single source of heat, by means of entropy generation.

The entropy generation associated with irreversible phenomena and based on the Second Law of Thermodynamics is of considerable importance in the fields of engineering sciences. Different phenomena give rise to entropy generation, such as heat transfer through finite temperature gradients, heat transfer characteristics, and effects of fluid viscosity. In recent years, the attention of researchers has increasingly focused on the utility of the concept of entropy generation in many applications, such as combustion engines and convection heat transfer systems. Bejan [2,3] conducted extensive theoretical work based on analysis, and minimized the entropy generation in fluid flow processes. This has shown that the entropy production rate can be used as an effective tool for the optimal design of thermal systems. Som and Datta [4] studied thermodynamic irreversibility in the combustion process of gaseous, liquid, and solid fuels. In a natural and mixed convection flow, Oztop and Al-Salem [5] studied entropy generation in enclosures. The important result learned from these works is that the main reasons for entropy generation are heat transfer and fluid friction. Ko and Wu [6] have focused on the effects of aspect ratios on entropy production induced by turbulent forced convection in rectangular ducts with curvature. They proposed an optimal aspect ratio based on the principle of minimizing entropy production. Gazzah and Belmabrouk [7,8] studied entropy generation in turbulent jets in the presence of co-flow. They found that the rate of total entropy production increases in three cases: The increase of the hot air jet temperature at the inlet, the decrease of the co-current velocity, and the increase of the deflection angles of the co-current. Moreover, the effects of a co-flowing stream on the mean and turbulent flow properties, mixing efficiency, air entrainment, entropy generation rate, and Merit and Bejan numbers of a heated turbulent plane jet emerging in a co-flowing stream have been investigated numerically by Elkaroui et al. [9], and they found that the co-flow has significantly reduced air entrainment. They also [10] examined the entropy generation of a turbulent plane jet with variable density, and found that the viscous dissipation was important near the nozzle exit and becomes negligible in the affinity region of the jet. Additionally, they show that the Merit number and the entropy generation rate increase gradually to stretch an asymptotic value along the flow direction as the inlet jet temperature increases. Mahmud et al. [11], Akih-Kumgeh [12], Kostic [13], Yilbas et al. [14], Shuja et al. [15], Demirel et al. [16], Bouras et al. [17,18], and Zimmermann et al. [19] conducted some very interesting studies on the second law and the generation of entropy responding to heat transfer and fluid friction in duct flows under different conditions. Entropy generation in combustion systems has only been a matter of attention within scientific fields. Dunbar and Lior [20] estimated the irreversible degree in methane and hydrogen premixed flames. Goodarzi et al. [21] investigated numerically the effect of radiation on laminar and turbulent mixed convection heat transfer, and the simulations exhibited that for a constant Richardson number, calculating the radiation heat transfer majorly affected the heat transfer structure in the enclosure; however, it’s impact on the fluid flow structure was negligible. Entropy generation has been examined by Chen et al. [22,23,24,25] differently than we have seen before in the literature. They found that the distribution of the entropy generation due to the fluid friction could not be ignored, and they concluded that the included entropy generation augmented proportionally with the addition of hydrogen. Today, combustion is one of the main means of converting energy. It is used in many practical systems, as well as for producing heat (boilers or furnaces, both domestic and industrial), electricity (thermal power plants), and for transport (automotive and aeronautical engines, rocket engines, etc.). In this work, we carry out a numerical simulation of the combustion of propane with air and reversibility (second law) analysis, by taking the effects of the gradient of temperature and velocity into account. The entropy generation number, irreversibility ratio (Bejan number), and variations of temperature distribution with turbulent flow parameters are illustrated graphically and discussed in the sections below.

## 2. Computational Model

**Figure 1:**
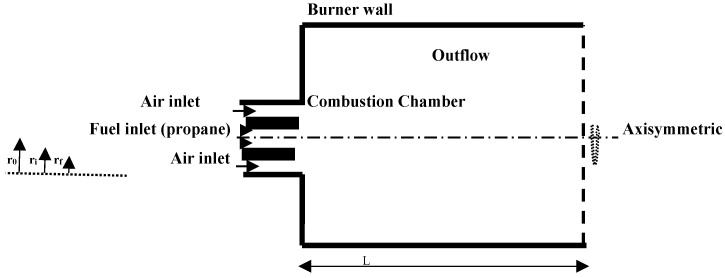
Geometry of the burner.

The purpose of this simulation is to evaluate numerically the rate of local entropy generation and the effect of different percentages of oxygen on the combustion of the propane air mixture in the enclosure of a burner. It was decided to solve the present problem with numerical simulation using Fluent 14.0 Computational fluid dynamics (CFD) code, an approach already submitted to verification and grid validation in previous works of the main authors for the same computational configuration used by Morsli et al. [26,27], which consists of a two-dimensional (2D) axisymmetric domain of the burner, as sketched in the Figure 1. As observed, fuel and air penetrated coaxially and merged downstream. It was supposed that the wall of the burner is submitted to ambient values, and that the walls close to the air and fuel inlets are insulated. The computational model employed the RNG (renormalization group theory) *k*–*ε* for turbulent flow. Nonetheless, for mass transfer (chemical species and reacting flow), the turbulent-dissipation model with a diffusion energy source is the respected option. The mixture (propane-air) was adopted as an ideal gas.

The length and the diameter of the combustion chamber were *L* = 0.5 m and *R* = 0.05 m, respectively. The diameters *r_f_, r_i_, r_o_* were of the order of 0.004, 0.006, and 0.01, respectively.

### 2.1. Mathematical Formulation

For the two-dimensional steady flow of an incompressible Newtonian fluid, balance equations give:

The conservation of mass and species transport is written as follows:

(1) $$\frac{\partial \rho }{\partial t}+\frac{\partial \rho u_{i}}{\partial x_{i}}=0$$

For *k* species it writes as

(2) $$\frac{\partial \rho Y_{k}}{\partial t}+\frac{\partial }{\partial x_{i}}(\rho (u_{i}+V_{k,j})Y_{k})={\dot{\omega }}_{k}$$

The momentum conservation gives

(3) $$\frac{\partial }{\partial t}\rho u_{j}+\frac{\partial }{\partial x_{i}}\rho u_{i}u_{j}=-\frac{\partial P}{\partial x_{i}}+\frac{\partial \tau_{i,j}}{\partial x_{i}}+\rho \sum_{k=1}^{N}Y_{k}f_{k,j}=\frac{\partial \sigma_{ij}}{\partial x_{i}}+\rho \sum_{k=1}^{N}Y_{k}f_{k,j}$$

and

(4) $$\tau_{ij}=-\frac{2}{3}\mu \frac{\partial u_{k}}{\partial x_{k}}\delta_{ij}+\mu \left(\frac{\partial u_{i}}{\partial x_{j}}+\frac{\partial u_{j}}{\partial x_{i}}\right)$$

The energy conservation is defined as

(5) $$\frac{\partial \rho H_{s}}{\partial t}+\frac{\partial }{\partial x_{i}}\left(\rho u_{i}h_{s}\right)={\dot{\omega }}_{T}+\dot{Q}+\frac{DP}{Dt}+\frac{\partial }{\partial x_{i}}(\lambda \frac{\partial T}{\partial x_{i}})+\tau_{ij}\frac{\partial u_{j}}{\partial x_{i}}-\frac{\partial }{\partial x_{i}}(\rho \sum_{k=1}^{N}V_{k,i}Y_{k}h_{s,k})$$

The RNG *k*-*ε* “turbulence model” leads to two additional expressions for the turbulence kinetic energy *k* and the dissipation rate *ε*, given respectively as follows:

$$\frac{\partial }{\partial x_{i}}\left(\rho x_{i}k\right)=\frac{\partial }{\partial x_{i}}(\alpha_{k}\mu_{eff}\frac{\partial k}{\partial x_{i}})+G_{k}-\rho \varepsilon$$

(6) $$\frac{\partial }{\partial x_{i}}\left(\rho x_{i}\varepsilon \right)=\frac{\partial }{\partial x_{i}}(\alpha_{\varepsilon }\mu_{eff}\frac{\partial k}{\partial x_{i}})+\frac{\varepsilon }{k}(C_{1\varepsilon }G_{k}-C_{2\varepsilon }\rho \varepsilon -\chi )$$

This mathematical model corresponds to the work of Magnussen and Hjertager [28], and is named the eddy-dissipation model. The species *i* production from the reaction *r* is *R_i,r_*, given as follows:

(7) $$R_{i,r}=v_{i,r}^{\prime }W_{\omega ,i}A\rho \frac{\varepsilon }{k}\min_{R}\left(\frac{Y_{R}}{v_{R,r}^{\prime }W_{\omega ,R}}\right)\;R_{i,r}=v_{i,r}^{\prime }W_{\omega ,i}AB\rho \frac{\varepsilon }{k}\left(\frac{\sum pY_{p}}{\sum_{J}^{N}v_{j,r}^{\prime \prime }W_{\omega ,j}}\right)$$

### 2.2. Combustion and Reaction Mechanism

The simplest representation of combustion is that it is a process that converts the reactants available at the creation of combustion into products at the end of the process. Commonly, combustion processes involved in engineering consist of converting a hydrocarbon fuel (which might range from pure hydrogen to almost pure carbon (*C*)—e.g., coal) into carbon dioxide and water. In this investigation, the combustion of fuel with propane is modeled with a one-step reaction mechanism (NR = 1). The reaction mechanism arises according to the constraints of chemistry, and is defined by [26]

(8) $$C_{3}H_{8}+\frac{5}{\phi }O_{2}+\frac{5}{\phi }\frac{100-\gamma }{\gamma }O_{2}\to 3CO_{2}+4H_{2}O+\frac{5}{\phi }\frac{100-\gamma }{\gamma }N_{2}+5\frac{100-\phi }{\phi }O_{2}$$

where

(9) $$\phi =5[Wo_{2}+(100-\gamma )/\gamma W_{N2}{\dot{m}}_{fuel}]/(W_{fuel}{\dot{m}}_{air})$$

In this work, the combustion of an equal composition hydrogen-propane mixture (50% H_2_:50% C_3_H_8_) is considered. It is established in Reference [29] that hydrogen reduces the emission of some unsafe components; pure hydrogen fuel combustion avoids CO or unburned HC production.

### 2.3. Entropy Generation Rate

In fluid flow, irreversibility takes place because of the heat transfer and the viscous effects of the fluid. In these systems, since heat and dynamic fields are fixed, the volumetric entropy generation rate $s_{gen}^{\prime \prime \prime }$ at every location in the system can be estimated as follows [2]:

(10) $$s_{gen}^{\prime \prime \prime }={\left(s_{gen}^{\prime \prime \prime }\right)}_{heat}+{\left(s_{gen}^{\prime \prime \prime }\right)}_{fric}$$

where ${\left(s_{gen}^{\prime \prime \prime }\right)}_{heat}$ and ${\left(s_{gen}^{\prime \prime \prime }\right)}_{fric}$ represent the entropy generation rates due to heat transfer and fluid friction, respectively, and are defined as

(11) $${\left(s_{gen}^{\prime \prime \prime }\right)}_{heat}=\frac{\lambda_{eff}}{T^{2}}\times \left[{\left(\frac{\partial T}{\partial x}\right)}^{2}+{\left(\frac{\partial T}{\partial r}\right)}^{2}\right]$$

and

(12) $${\left(s_{gen}^{\prime \prime \prime }\right)}_{fric}=\frac{\mu_{eff}}{T}\times \Phi$$

where Φ is

$$\Phi =2\times \left[{\left(\frac{\partial U_{x}}{\partial x}\right)}^{2}+{\left(\frac{\partial U_{r}}{\partial r}\right)}^{2}+{\left(\frac{U_{r}}{r}\right)}^{2}\right]+{\left(\frac{\partial U_{x}}{\partial r}+\frac{\partial U_{r}}{\partial x}\right)}^{2}$$

The total entropy generation rate over the volume ${\overset{•}{S}}_{gen}$ can be calculated as follows:

(13) $${\overset{•}{S}}_{gen}=\oint {S^{\prime \prime \prime }}_{gen}d\theta \times dr\times dx$$

The Bejan number *Be* is defined by

(14) $$Be={\left({\overset{•}{S}}_{gen}\right)}_{heat}/{\overset{•}{S}}_{gen}$$

Values of Be〉〉0.5 refer to heat transfer irreversibility dominating, while values of Be〈〈0.5 refer to viscous effects irreversibility dominating. For Be≅0.5, entropy generation induced heat transfer balances with induced fluid friction.

### 2.4. Numerical Tools

Fluent industrial software [30] was chosen as the CFD computational package, and is based on a finite-volume procedure to produce a numerical solution of the Navier–Stokes set of fluid flow in primitive variables (P, V, T). The code also provides solutions for transport of chemical components by solving partial differential equations describing basic mechanisms as convection, diffusion, and reaction for each constituent. The RNG model was chosen for turbulence modeling [31], while velocities of the entrance of air, and stoichiometric air/fuel ratios, are listed in the Table 1. The model is axisymmetric. The second-order upwind scheme was chosen for discretization to ensure higher-order precision at cell faces.

## 3. Simulation Results

### 3.1. Operating Values and Validation

The propane-air mixture had the following physical properties:$T_{in}=T_{amb}=T_{ref}=300\;K$, ϕ, $\rho_{air}=1.2555\;\mathrm{Kg}/m^{3}$, and $\rho_{C_{3}H_{8}}=1.91\;\mathrm{Kg}/m^{3}$ at the air and fuel entrance. The propane density at the fuel inlet, and the molecular weights, enthalpies, and lower heating values of the reactant and product species, were taken from the Fluent Inc. material property database given in Reference [30].

The equivalence ratio ϕ is defined as [32]

(15) $$\phi =\frac{\left(Y_{F}/Y_{O}\right)}{{\left(Y_{F}/Y_{O}\right)}_{st}}$$

where *Y_F_* and *Y_O_* represent the fuel and oxidizer mass fractions, respectively. The subscript *st* refers to stoichiometric conditions.

In this work, the total cell number of 15,000 cells was adopted. Four other grids have been tested, and the grid mesh used guaranteed a good compromise between physical results, grid sensitivity, and computational results. To reach the validation target, the predicted wall temperature obtained from the numerical results was compared with the available numerical data from the literature [33]. The temperature and reaction rates were less than 2% and 1%, respectively.

### 3.2. Physical Fields

#### 3.2.1. Reaction Rates

A chemical combustion process is a succession of cuts and creations of bonds between molecules. Each cut and creation of a bond is an elementary reaction, and the set of elementary reactions constitutes the kinetic mechanism of the reaction process. The final state of the reaction process corresponds to the chemical equilibrium; the composition of the mixture is then defined by the laws of thermodynamics.

Depending on the temperature and pressure conditions of the final state, chemical species appear or disappear. Some species produced, even in very small proportions, are of great importance, as is the case with certain polluting species (nitrogen oxide, carbon monoxide, etc.).

**Figure 2:**
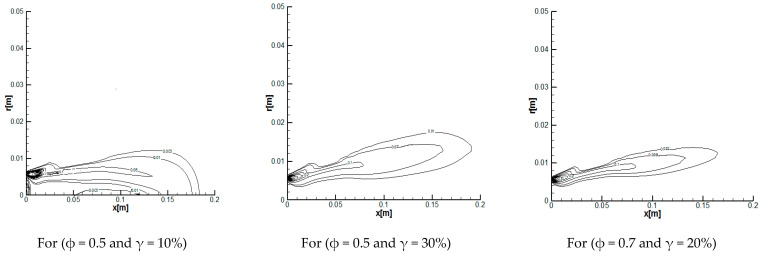

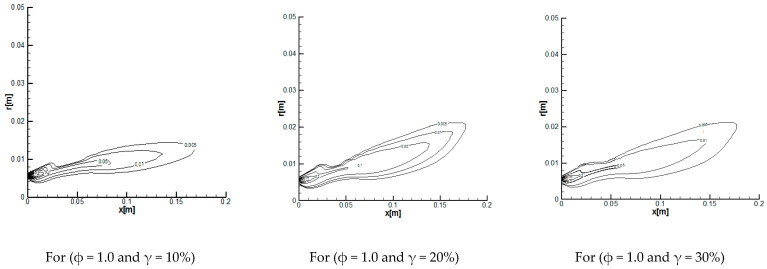
Contours of reaction rates for different values of the equivalence ratio ϕ and oxygen percentage in air.

In Figure 2, the propane-air flame is simulated for the distributions of reaction rates in the combustion chamber for different oxygen percentages and three equivalence ratios *φ* = 0.5, 0.7, and 1. One notices the presence of a large toric vortex, which forms a zone of recirculation. This is generated by the form of the combustion chamber [34], which has a sudden enlargement, and initiates a deflection of the flow in the reaction zone. It has been found that with the increase in the percentage of oxygen *γ* from 10% to 30%, the reaction rates decrease in the axial direction of the burner, whereas they increase in the radial direction. As shown at these figures, as the values of *φ* decrease (or the values of *λ* increase), combustion starts earlier. However, the reaction rate levels decrease significantly with the increase of both the equivalence ratio φ and the oxygen percentage *λ* [35].

#### 3.2.2. Temperature Distribution

**Figure 3:**
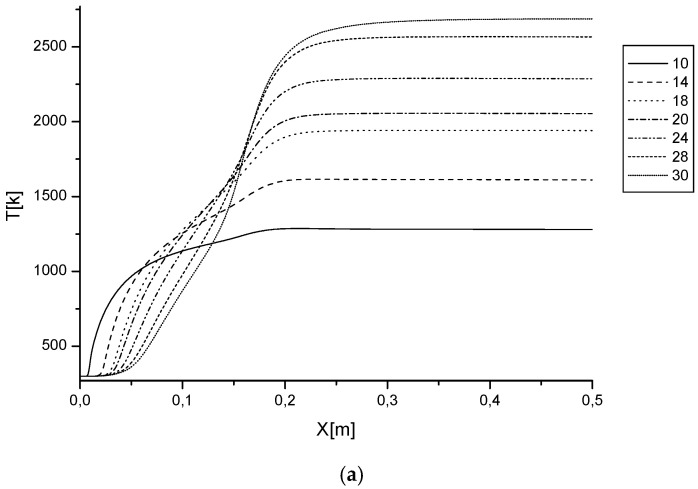

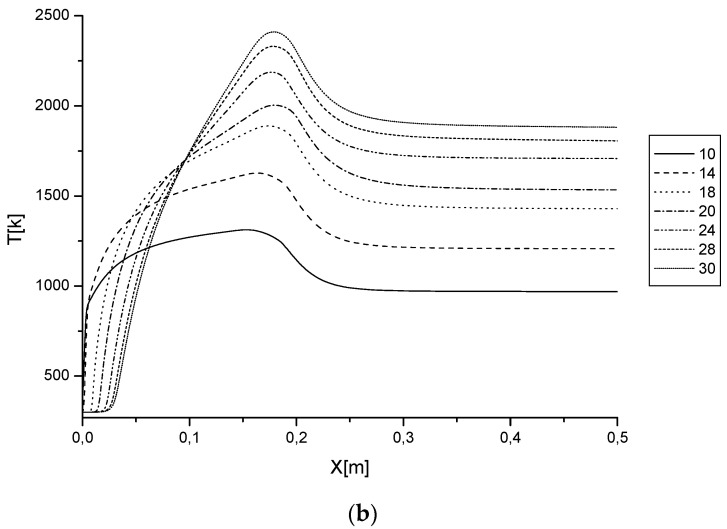
Variations of temperature at the axis of the burner depending on the axial distance for (**a**) ϕ = 0.5 and (**b**) ϕ = 1.0, at different values of γ.

In order to follow the behavior of these temperature gradients in the axial direction, it was necessary to plot the variation of the latter along the axis of the burner. Figure 3 illustrates the variations in temperature along the axis of the burner. It is noted that between *x* = 0 and *x* = 0.2, large temperature gradients are present. From the figure, it can be seen that the increase of φ from 0.5 to 1 significantly reduces the temperature profiles. Although, the increase of γ (10 to 30%) considerably increases the temperature profiles. The maximum combustion temperature is very high and reaches a value of 2500 K. This requires taking into account the effect of these parameters on the construction of the combustion chamber wall. Feeding the flame on propane input led to an increase in the overall temperature of the flame, reducing CO and unburned HC emissions. NO emission through the thermal mechanism arises.

#### 3.2.3. Entropy Generation and Bejan Number

**Figure 4:**
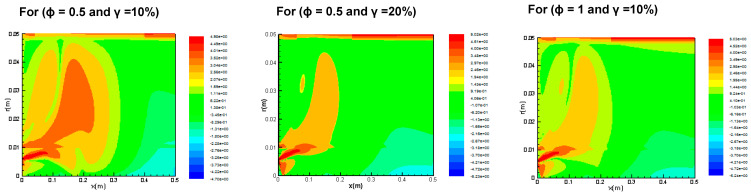

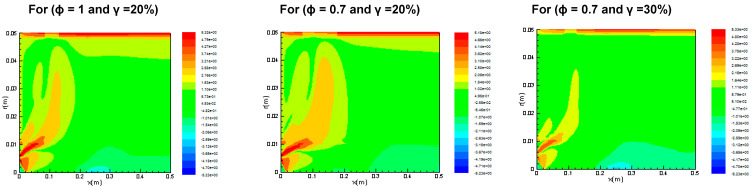
Logarithmic volumetric local entropy generation rate contours for ϕ = 0.5, 0.7, and 1.0, at different values of γ.

Figure 4 indicates the contours of the logarithmic volumetric entropy generation rate for different equivalence ratios (φ = 0.5, 0.7, and 1.0) and different percentages of oxygen (γ = 10, 20, and 30%). The results show that the generation of entropy increases in locations where reactions are effective, then the temperature shows strong variation. The volumetric local entropy generation rates reduce by about 10 and 5% in the cases of φ = 0.5, 0.7, and 1, with a respective increase of γ from 10 to 20 and 30%. Logarithmic entropy generation rates due to fluid friction are much lower than those due to heat transfer. This introduces heat transfer irreversibility.

The contours of the generation of entropy do not allow us to show which of the viscous and thermal effects dominate. The Bejan number can better explain this situation. This number makes it possible to show the contribution of thermal effects in the total generation of entropy. It is defined as the ratio of the thermal generated entropy to the total generated entropy.

The Bejan number ranges from 0 to 1. Consequently, if it is equal to 1, the term relating to the thermal effects dominates; if it is equal to 0, the term relating to viscous friction dominates; and if it is equal to ½, the contribution of the two terms is equal.

**Figure 5:**
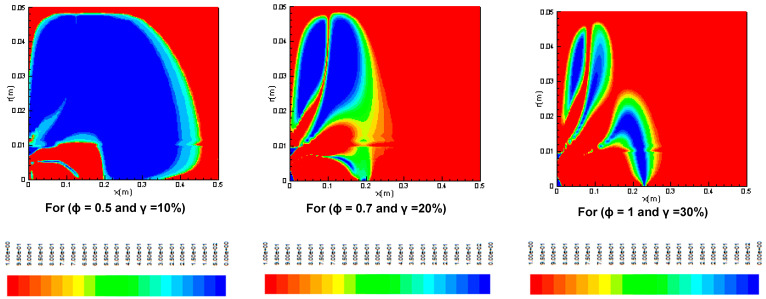
Bejan Number Contours.

Figure 5 shows the influence of the equivalence ratios φ = 0.5, 0.7, and 1, and the percentage of oxygen γ ranging from 10% to 30%, on the Bejan number. In any case, a concentration of the Bejan number near the walls indicates the high entropy generation region. This means that the generation of entropy is entirely dominated by heat transfer.

## 4. Conclusions

In this work, the combustion of propane with air in a burner was carried out to determine numerically the local entropy generation rate in a combustion chamber. The effects of the oxygen fraction in the air and the equivalence ratio on combustion generation were also studied. The governing equations were solved by the finite volume method under the commercial CFD Fluent code. The effects of turbulence were modeled by the RNG-K-epsilon model, while the entropy generation was introduced in post-processing. The entropy generation rates due to heat transfer and the viscous friction of the fluid were computed. The main conclusion is that increases in the equivalence ratio remarkably minimize the reaction rate levels and cause a delayed start to combustion. This result corroborates with the available published ones.

In the case when the quantity of air available for combustion is greater than the chemical quantity for complete oxidation of the propane (excess of air available), complete combustion occurs, and when the quantity of air available for combustion is equal to the chemical quantity for complete oxidation of the propane, the combustion is very close to the complete state.

The highest temperatures in the combustion enclosure augment through the amount of oxygen in air (from 10 to 30%) and the equivalence ratio (from 0.5 to 1.0).

The increase in temperature increases thermo-mechanical irreversibility and decreases chemical exergy with an increase in the oxygen fraction in air. The results show that the oxygen fraction in air has a greater effect on temperature than the equivalence ratio.

In all cases studied, the Bejan number measuring the contribution of the heat exchange to the entropy generation is very close to 1 (about 0.994). Consequently, heat transfer irreversibility dominates.

Hydrogen mixed with propane leads to considerable diminution in temperature levels, and a consequent expected reduction in CO and unburned HC; however, higher NOx emanations are expected [33].

Future work will focus on providing better understanding through using the entropy generation approach on heat and fluids flowing in combustion chambers. Other mixtures will be tested.

## Figures and Tables

**Table 1:** Inlet velocities of air for *U_f_* = 2.247 m/s and $\dot{\;Q}=1000$ W.

U Air (m/s)			
λ(%)	*ϕ* = 0.5	*ϕ* = 0.7	*ϕ* = 1
10	56.436	40.312	20.218
20	28.614	20.439	14.307
30	19.340	13.814	9.670

## Nomenclature

**Latin Symbols**	
*Be*	Bejan number
CFD	Computational fluid dynamics
Cμ, Cε1, Cε2	Coefficients in *k*–*ε* turbulence model
*f_k,j_*	Specific force of volume acting on the species *K* in the direction i
*H*	Enthalpy
h_k_	Specific enthalpy of the species *k*
h_s,k_	Heat transfer coefficient, sensible enthalpy of species
K	Turbulent kinetic energy
*L*	Length of burner
LCV	Lower calorific value
P	Pressure
RNG	Renormalization group
R	Radial distance
$r_{i}$	Inner radius of air inlet
$r_{0}$	Outer radius of air inlet
$s_{gen}^{\prime \prime \prime }$	Volumetric entropy generation rate
T	Temperature
u_i_	Velocity in direction *i*
V_k,i_	Velocity diffusion of the species *k* in direction *i*
Y_k_	Mass fraction of the species *k*
**Greek Symbols**	
$\delta_{ij}$	Kronecker delta
ε	Turbulent energy dissipation rate
ϕ	Equivalence ratio
Φ	Viscous dissipation
λ	Air excess ratio
μ	Dynamic viscosity
ρ	Density
θ	Tangential direction
$\rho_{k}$	Density of the species *k*
$\sigma_{ij}$	Tensor of the constraint in plan *i* and the direction *j*
${\dot{\omega }}_{k}$	Production rate of the *k* species.
$\dot{Q}$	Heat transfer rate
$\tau_{ij}$	Tensor of the viscous constraints
**Subscripts**	
F	Fuel
K	Species
N	Total number of species number
O	Oxidant
P	At constant pressure
S	Sensibility (enthalpy)
V	At constant volume
R	Radial
X	Axial
Amb	Ambient
Air	Air
Eff	Effective
Fric	Friction
Heat	Heat transfer
i,j	Indices of tensor notation
In	Inlet
